# A model combining rest-only ECG-gated SPECT myocardial perfusion imaging and cardiovascular risk factors can effectively predict obstructive coronary artery disease

**DOI:** 10.1186/s12872-022-02712-8

**Published:** 2022-06-15

**Authors:** Bao Liu, Wenji Yu, Jianfeng Wang, Xiaoliang Shao, Feifei Zhang, Mingge Zhou, Yunmei Shi, Bing Wang, Yiduo Xu, Yuetao Wang

**Affiliations:** 1 Department of Nuclear Medicine, The Third Affiliated Hospital of Soochow University, No.185, Juqian Street, Changzhou, 213003 Jiangsu Province China; 2 The Nuclear Medicine and Molecular Imaging Clinical Translation Institute of Soochow University, Changzhou, Jiangsu Province China

**Keywords:** Myocardial perfusion imaging, SPECT, Prediction model, Coronary artery disease

## Abstract

**Objective:**

The rest-only single photon emission computerized tomography (SPECT) myocardial perfusion imaging (MPI) had low sensitivity in diagnosing obstructive coronary artery disease (CAD). Improving the efficacy of resting MPI in diagnosing CAD has important clinical significance for patients with contraindications to stress. The purpose of this study was to develop and validate a model predicting obstructive CAD in suspected CAD patients, based on rest-only MPI and cardiovascular risk factors.

**Methods:**

A consecutive retrospective cohort of 260 suspected CAD patients who underwent rest-only gated SPECT MPI and coronary angiography was constructed. All enrolled patients had stress MPI contraindications. Clinical data such as age and gender were collected. Automated quantitative analysis software QPS and QGS were used to evaluate myocardial perfusion and function parameters. The least absolute shrinkage and selection operator (LASSO) and multivariable logistic regression were used to select the variables and build the prediction model.

**Results:**

Among the enrolled 260 patients with suspected CAD, there were 95 (36.5%, 95/260) patients with obstructive CAD. The prediction model was presented in the form of a nomogram and developed based on selected predictors, including age, sex, SRS ≥ 4, SMS ≥ 2, STS ≥ 2, hypertension, diabetes, and hyperlipidemia. The AUC of the prediction model was 0.795 (95% CI: 0.741–0.843), which was better than the traditional models. The AUC calculated by enhanced bootstrapping validation (500 bootstrap resamples) was 0.785. Subsequently, the calibration curve (intercept = − 0.106; slope = 0.843) showed a good calibration of the model. The decision curve analysis (DCA) shows that the constructed clinical prediction model had good clinical applications.

**Conclusions:**

In patients with suspected CAD and contraindications to stress MPI, a prediction model based on rest-only ECG-gated SPECT MPI and cardiovascular risk factors have been developed and validated to predict obstructive CAD effectively.

## Introduction

Coronary artery disease (CAD) seriously endangers human health in China and the world. Coronary stenosis ≥ 70% based on coronary angiography is defined as obstructive CAD. Notably, in the narrow coronary arteries > 70%, more than 80% have functional myocardial ischemia [1]. Single photon emission computerized tomography (SPECT) myocardial perfusion imaging (MPI) is a widely used non-invasive method for detecting myocardial ischemia and CAD [2, 3]. Abnormal MPI under stress and partial or complete restoration at rest are considered as typical manifestations of myocardial ischemia. In general, the sensitivity of exercise stress SPECT MPI for the diagnosis of CAD was 82–88%, and the specificity was 70–88%; the sensitivity of pharmacologic stress SPECT MPI for the diagnosis of CAD was 88–91%, and the specificity was 75–90% [4].

However, in patients with suspected acute coronary syndrome (ACS) and severe heart failure, stress MPI was contraindicated [5]. Meanwhile, previous study had found that the sensitivity of resting perfusion abnormalities for diagnosing CAD was low, at approximately 30% [6]. Despite the addition of wall motion, the sensitivity of resting MPI is only 46.8% [7], which is still low. Electrocardiogram (ECG)-gated SPECT MPI can provide additional information on global and regional myocardial function beyond perfusion [8–10], offering the opportunity for multiparametric modelling. Model based on artificial intelligence algorithms have also been explored in stress or stress/rest MPI for the diagnosis of obstructive CAD [11]. However, there are few studies on the modeling of patients undergoing rest-only gated MPI.

Therefore, based on relevant research and our preliminary analysis of data and clinical experience, we have developed and verified a nomogram for predicting the risk of obstructive CAD through rest-only ECG-gated MPI and clinical parameters. The proposed predictive model may help improve the efficacy of rest-only ECG-gated MPI in diagnosing obstructive CAD for early intervention and treatment.

## Methods

### Study cohort and population

**Fig. 1:**
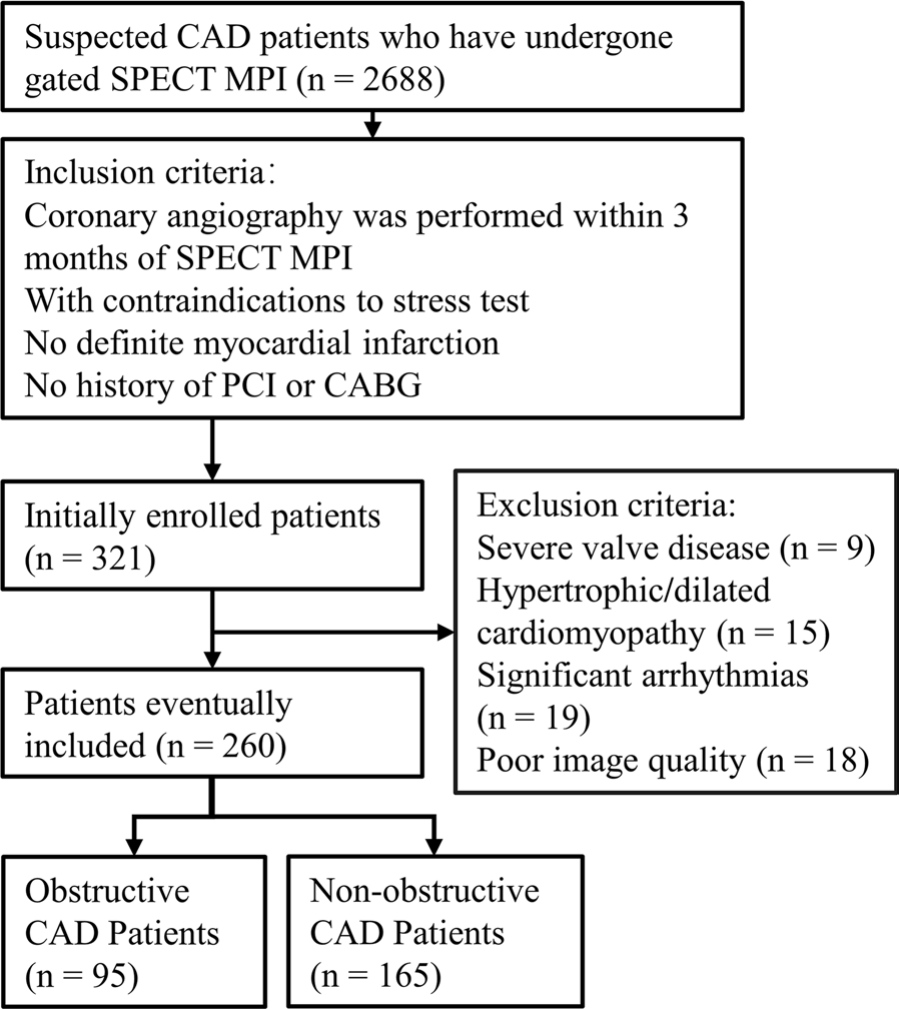
Flow chart of patient enrollment. *CAD* coronary artery disease, *SPECT* single photon emission computerized tomography, *MPI* myocardial perfusion imaging, *PCI* percutaneous coronary intervention, *CABG* coronary artery bypass grafting

This is a retrospective single-center cohort study of patients who underwent ECG-gated SPECT MPI for suspected CAD at the Third Affiliated Hospital of Soochow University between February 2016 and April 2020. Inclusion criteria included coronary angiography performed within 3 months of SPECT MPI, with contraindications to stress test, without history of myocardial infarction, percutaneous coronary intervention (PCI) or coronary artery bypass grafting (CABG). Three hundred and twenty-one patients were initially enrolled. Exclusion criteria were as follows: (1) severe valve disease (n = 9), (2) hypertrophic or dilated cardiomyopathy (n = 15), (3) significant arrhythmias (beat rejection > 5%) (n = 19), (4) poor quality of raw images that were difficult to analyze (n = 18). Finally, 63 patients with suspected ACS, 39 patients with decompensated heart failure, 42 patients with bradycardia combined with impaired physical activity, 55 patients with uncontrollable hypertension (systolic blood pressure > 200 mmHg) and 61 asthmatics were included in the final study, for a total of 260 patients. None of them could perform stress test in our center. The flow chart of patient enrolled is shown in Fig. 1. The study protocol was in accordance with the Declaration of Helsinki and was approved by the ethics committee of the Third Affiliated Hospital of Soochow University.

### Resting image acquisition

Resting image acquisition meets the recommendations of relevant guidelines [12]. ECG-gated SPECT MPI was initiated 60–90 min after intravenous injection of ^99m^technetium-sestamibi (^99m^Tc-MIBI) (740–925 MBq) at rest. A dual-head 90° gamma camera (Symbia T16, Siemens Medical Systems, Erlangen, Germany) equipped with a parallel-hole collimator with low energy and high resolution was used for image acquisition. The acquisition energy window was 140 ± 20% KeV. Sixty-four images covering 180° were obtained using a 64 × 64 matrix and a magnification of 1.45, with 8 frames per R-R cycle. The projection data were filtered using a Butterworth filter (order, 5; cutoff frequency, 0.4) and then reoriented by filtered back projection to obtain left ventricular (LV) short-axis, horizontal long-axis, and vertical long-axis images. The attenuation correction was not used in this study.

### Quality control

Strict quality control was performed during raw image acquisition, reconstruction and image quantification. During image acquisition, we checked whether myocardial radioactivity uptake was good to avoid leakage of imaging agent injections, and radioactive focus outside the heart. For data with significant movement, a re-acquisition procedure was performed. We also checked the gated quality control curve, sinogram and linogram in the tomographic processing. Patients who did not meet the above quality control requirements were not included in this study. Image quantification was performed automatically by the software. When the automatic delineation was poor, we would make appropriate manual adjustments to ensure accurate sampling of the myocardium boundary contours.

### Resting image analysis

A 17-segment model of the LV was used for image analysis. As previously described [13], the 17 segments were divided into the territories of left anterior descending coronary artery (LAD), left circumflex coronary artery (LCX) and right coronary artery (RCA). QPS and QGS 2009 software packages (Cedars-Sinai Medical Center, Los Angeles, CA, USA) were used for semi-quantitative analysis of perfusion and myocardial function. According to the degree of abnormality, the software automatically scores perfusion (0–4), wall motion (0–5) and wall thickening (0–3) abnormalities. The meaning of the scoring value can refer to related research [14, 15]. The summed rest score (SRS), summed motion score (SMS) and summed thickening score (STS) were the sum of all perfusion, wall motion or wall thickening abnormality scores on the rest scan respectively.

### Predictors and outcomes

Patients’ data were retrospectively obtained from electronic medical records. Based on clinical experience and actual situation, we finally collected the following 14 variables as initial predictors, including demographic information (age, sex, body mass index (BMI)), traditional risk factors (hypertension, diabetes, hyperlipidemia, smoking history), perfusion information (SRS) and myocardial functional parameters (end-diastolic volume (EDV), end-systolic volume (ESV), left ventricular ejection fraction (LVEF), peak filling rate (PFR), SMS, STS). Hypertension was defined as a systolic blood pressure > 140 mmHg or currently being treated with antihypertensive drugs. Patients with a previous diagnosis or currently treated with hypoglycemic medications were considered to have diabetes. Hyperlipidemia was defined as a confirmed history of hyperlipidemia or current treatment with lipid-lowering medication [16]. In this study, SRS ≥ 4 exhibited in 2 consecutive segments (each up to grade 2) in one territory was considered abnormal [6]. SMS ≥ 2 or STS ≥ 2 exhibited in 2 consecutive segments in one territory was considered abnormal [14].

All coronary angiograms were visually interpreted by 2 or more experienced cardiologists. Obstructive CAD was defined as ≥ 70% narrowing of the inner diameter of the LAD, LCX, RCA or their main branches and ≥ 50% narrowing of the left main coronary artery [17].

### Statistical methods

All Statistical analysis was performed using the R statistical software (R version 4.1.0). Continuous variables conforming to the normal distribution were represented by the mean ± SD, and continuous variables that do not conform were expressed by the median P50 (P25, P75). The baseline characteristics between groups were compared using the Unpaired t test or Mann–Whitney U test for continuous variables, chi-square tests for categorical variables where appropriate. The least absolute shrinkage and selection operator (LASSO) was used for variables filtering and selection. LASSO is more appropriate than traditional stepwise regression for small datasets with a low events per variables (EPV) ratios [18] and is suitable for regression models with high-dimensional predictor variables [19]. The final prediction model was constructed from a logistic regression model where the predictors were identified from the LASSO regression and presented as a nomograph.

The area under the receiver-operator characteristic curve (AUC) was plotted by MedCalc software package (Version 18.9.1) to assess the discrimination of the model. As the AUC gets closer to 1, the better the discrimination of the model. A value of 0.5 indicates that the model is equivalent to random sampling. The best cut-off value was determined using Youden index. The calibration curve, expressed as calibration intercept and slope, was used to evaluate the calibration of the model. Perfect calibration is indicated when the calibration intercept is 0 and the calibration slope is 1. Enhanced bootstrapping validation (500 bootstrap resamples) was used for internal validation. *P* < 0.05 was considered to be statistically significant.

## Results

### Patient characteristics

This study finally included 260 suspected CAD patients for analysis. Subsequent coronary angiography confirmed that 95 (36.5%, 95/260) patients had obstructive CAD (stenosis ≥ 70%). The median age of the obstructive CAD group was (62.7 ± 9.0 years), slightly higher than non-obstructive CAD group (60.8 ± 9.2 years, *P* = 0.101). In total, 89 (34.2%, 89/260) were female and 171 (65.8%, 171/260) were male. There were no significant differences in BMI between obstructive CAD group and without obstructive CAD group (24.6 ± 2.9 vs. 25.1 ± 3.0, *P* = 0.269). No significant difference in smoking history was observed between the two groups, but comorbidities, including hypertension, diabetes and hyperlipidemia, were more common in obstructive CAD compared to the group without obstructive CAD. Subjects with obstructive CAD had higher EDV, ESV and lower LVEF. Myocardial diastolic function, expressed by PFR, was significantly worse in the obstructive CAD group (2.3 ± 0.5 vs. 2.1 ± 0.6, *P* = 0.001), compared to the non-obstructive CAD group. Baseline characteristics of all patients were displayed in Table 1.

**Table 1:** Characteristics of patients with suspected CAD (n = 260)

Variables	Without obstructive CAD (n = 165)	With obstructive CAD (n = 95)	*P* value
Age (years old)	60.8 ± 9.2	62.7 ± 9.0	0.101
Male (%)	99 (60.0)	72 (75.8)	0.010
BMI (kg/m^2^)	25.1 ± 3.0	24.6 ± 2.9	0.269
Hypertension (%)	103 (62.4)	73 (76.8)	0.017
Diabetes (%)	26 (15.8)	35 (36.8)	< 0.001
Hyperlipidemia (%)	98 (59.4)	74 (77.9)	0.002
Smoking > 1 year (%)	59 (35.8)	41 (43.2)	0.238
Gated SPECT			
EDV (ml)	86.9 ± 25.2	100.4 ± 46.9	0.010
ESV (ml)	31.4 ± 14.8	46.3 ± 39.0	0.001
LVEF (%)	65.4 ± 8.5	58.3 ± 12.4	< 0.001
SRS ≥ 4 (%)	7 (4.2)	33 (34.7)	< 0.001
SMS ≥ 2 (%)	6 (3.6)	39 (41.1)	< 0.001
STS ≥ 2 (%)	6 (3.6)	34 (35.8)	< 0.001
PFR	2.3 ± 0.5	2.1 ± 0.6	0.001

*CAD* coronary artery disease, *BMI* body mass index, *SPECT* single photon emission computerized tomography, *EDV* end diastolic volume, *ESV* end systolic volume, *LVEF* left ventricular ejection fraction, *SRS* summed rest score, *SMS* summed motion score, *STS* summed thickening score, *PFR* peak filling rate

### Development of the prediction model

**Fig. 2:**
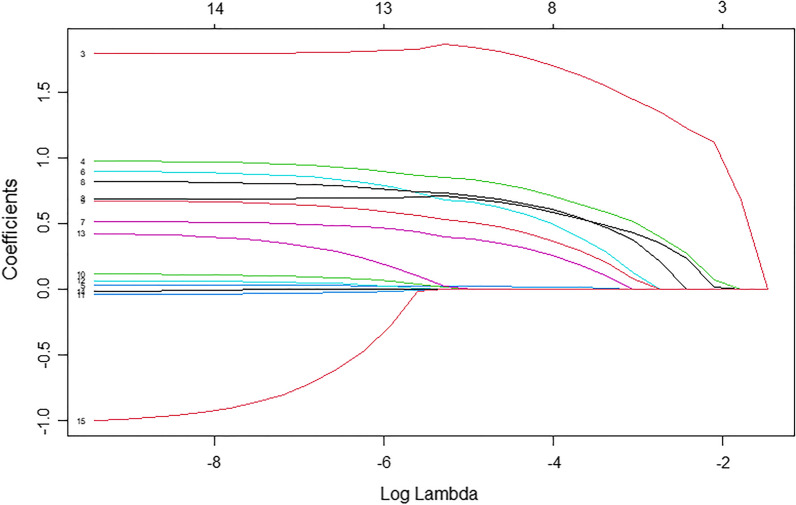
LASSO coefficient profiles of the selected features. A coefficient profile plot was produced against the log(λ) sequence, the optimal lambda resulted in eight features with nonzero coefficients

**Fig. 3:**
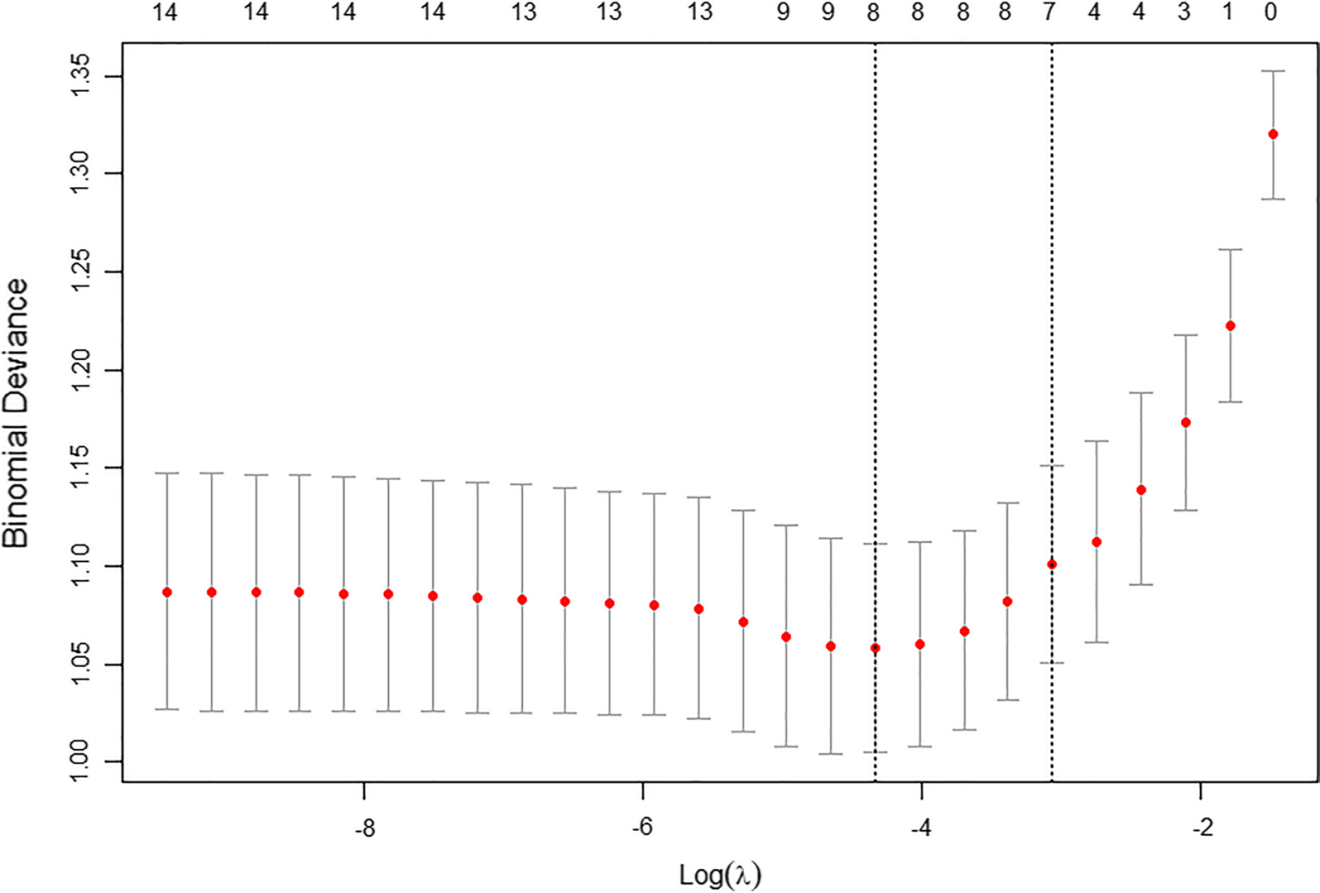
Selection of the optimal variable (λ) in the LASSO regression model used cross-validation via the minimum criteria. The partial likelihood deviance curve was plotted versus log(λ)

A total of 14 variables were included in the LASSO regression analysis. The following 8 variables with nonzero coefficients were selected by LASSO regression as predictors: age, sex, SRS ≥ 4, SMS ≥ 2, STS ≥ 2, hypertension, diabetes, and hyperlipidemia. The process of LASSO regression selection was shown in Figs. 2 and 3. According to the reported studies and clinical experience, these eight predictors were finally incorporated into logistic regression to construct a prediction model. In addition, the OR values of categorical variables were calculated from state 1 (positive) compared to state 0 (negative). The OR and *P* values of these 8 variables were as follows: age (OR, 1.03; 95% CI, 1.00–1.07; *P* = 0.092), sex (OR, 2.23; 95% CI, 1.15–4.51; *P* = 0.020), SRS ≥ 4 (OR, 2.21; 95% CI, 0.69–7.09; *P* = 0.200), SMS ≥ 2 (OR, 7.17; 95% CI, 2.20–26.0; *P* = 0.001), STS ≥ 2 (OR, 2.58; 95% CI, 0.75–9.11; *P* = 0.130), hypertension (OR, 1.61; 95% CI, 0.82–3.27; *P* = 0.200), diabetes (OR, 2.20; 95% CI, 1.07–4.52; *P* = 0.031), hyperlipidemia (OR, 1.85; 95% CI, 0.96–3.63; *P* = 0.069), as shown in Table 2.

**Table 2:** Logistic regression analysis of factors obtained by LASSO regression analysis

Variables	Regression coefficient	OR	95% CI	*P* value
Age	0.030	1.03	1.00–1.07	0.092
Male	0.804	2.23	1.15–4.51	0.020
Hypertension	0.477	1.61	0.82–3.27	0.200
Diabetes	0.790	2.20	1.07–4.52	0.031
Hyperlipidemia	0.613	1.85	0.96–3.63	0.069
SRS ≥ 4	0.794	2.21	0.69–7.09	0.200
SMS ≥ 2	1.971	7.17	2.20–26.0	0.001
STS ≥ 2	0.950	2.58	0.75–9.11	0.130

*SRS* summed rest score, *SMS* summed motion score, *STS* summed thickening score; the OR values of categorical variables were calculated from state 1 (positive) compared to state 0 (negative)

### Model performance and comparison

**Fig. 4:**
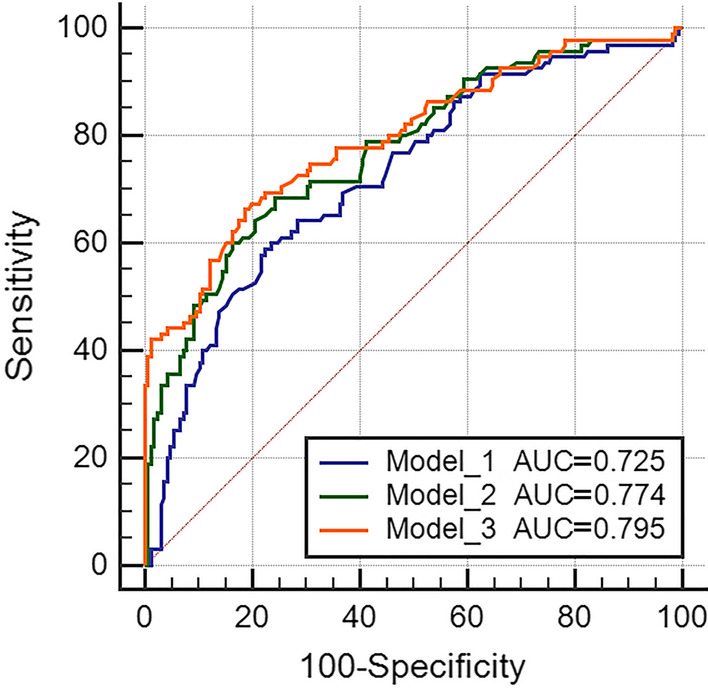
ROC analysis of constructed models and traditional models. *ROC* receiver operator characteristic curve, *AUC* the area under the receiver operator characteristic curve. Model_1: Traditional cardiovascular risk factors (including age, gender, hyperlipidemia, hypertension, diabetes); Model_2: Model_1 + SRS ≥ 4; Model_3: Model_2 + SMS ≥ 2 + STS ≥ 2

To demonstrate the advantages of this model, we built three models: Model_1, traditional cardiovascular risk factors (including age, gender, hyperlipidemia, hypertension, diabetes); Model_2, Model_1 + SRS ≥ 4; Model_3, Model_2 + SMS ≥ 2 + STS ≥ 2. The discrimination of the model was measured by AUC. The AUC for Model_1 was 0.725 (95% CI: 0.666–0.778, *P* < 0.001), Model_2 was 0.774 (95% CI: 0.718–0.823, *P* < 0.001), Model_3 was 0.795 (95% CI: 0.741–0.843, *P* < 0.001), as shown in Fig. 4.

**Fig. 5:**
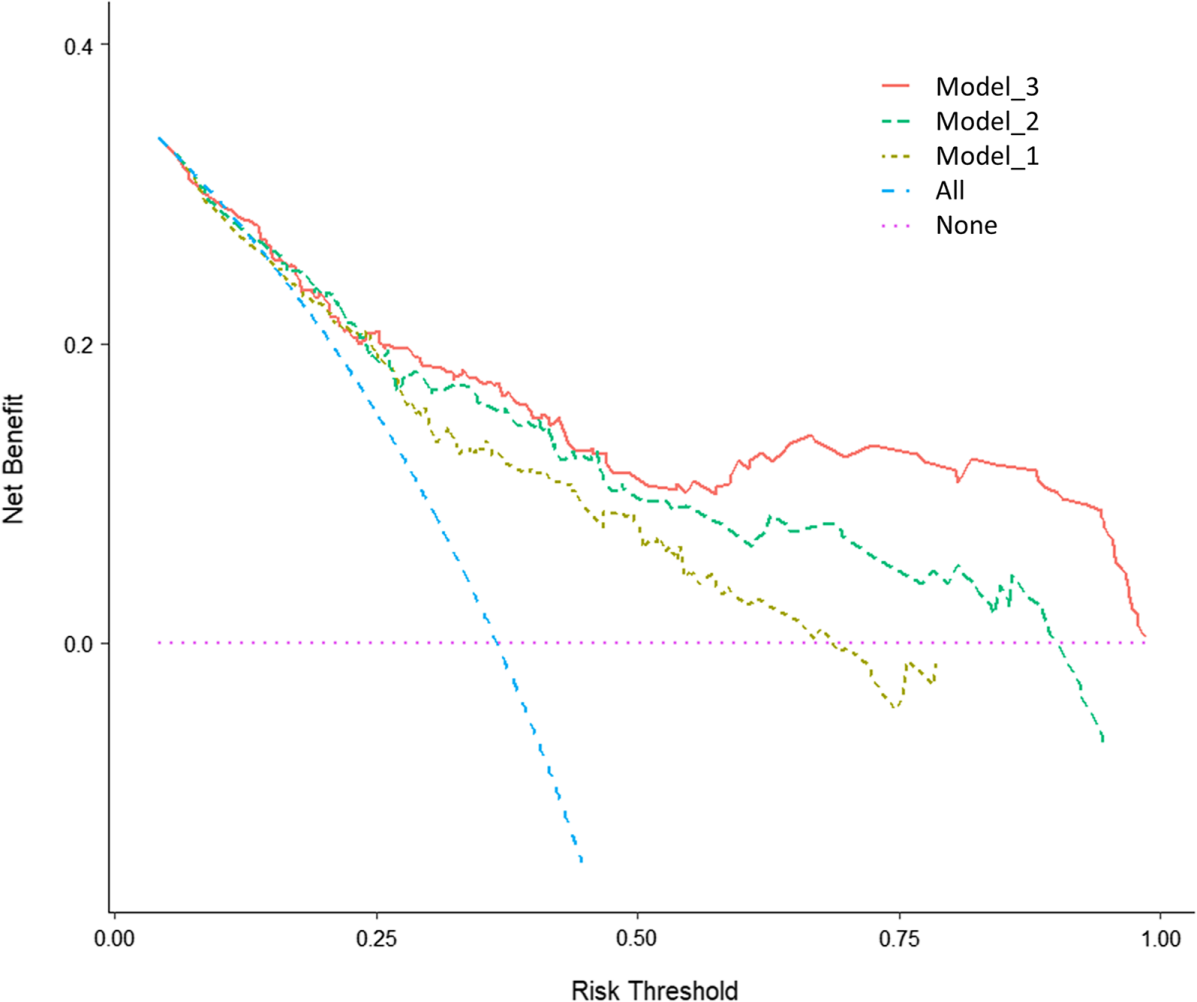
Decision curve analysis of the constructed model versus the traditional models. Model_1: Traditional cardiovascular risk factors (including age, gender, hyperlipidemia, hypertension, diabetes); Model_2: Model_1 + SRS ≥ 4; Model_3: Model_2 + SMS ≥ 2 + STS ≥ 2

**Fig. 6:**
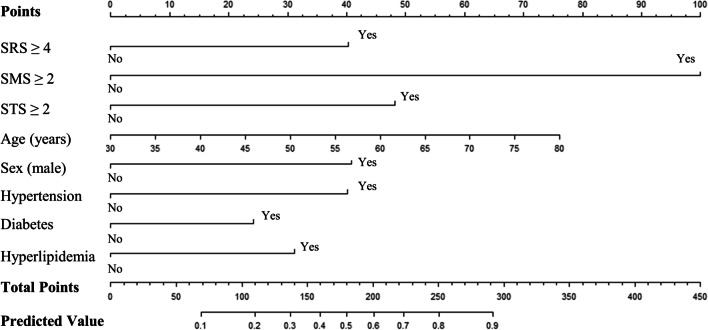
The nomogram for predicting patients with obstructive CAD based on eight independent diagnostic factors. *CAD* coronary artery disease, *SRS* summed rest score, *SMS* summed motion score, *STS* summed thickening score

The AUC of Model_3 was higher than Model_1 (0.795 vs. 0.725, *P* = 0.005), and Model_2 (0.795 vs. 0.774, *P* = 0.195). The cut-off value for Model_3 was 0.363, with sensitivity of 66.3% and specificity of 81.2%. The results of decision curve analysis (DCA) were shown in Fig. 5, and the Model_3 demonstrated good clinical application compared with Model_1 and Model_2. We created a nomogram based on Model_3 to predict the probability of obstructive CAD in patients with suspected CAD (Fig. 6). By calculating the sum of the scores assigned to each variable in the nomogram, the higher the total score, the higher the probability of obstructive CAD.

### Model internal validation

**Fig. 7:**
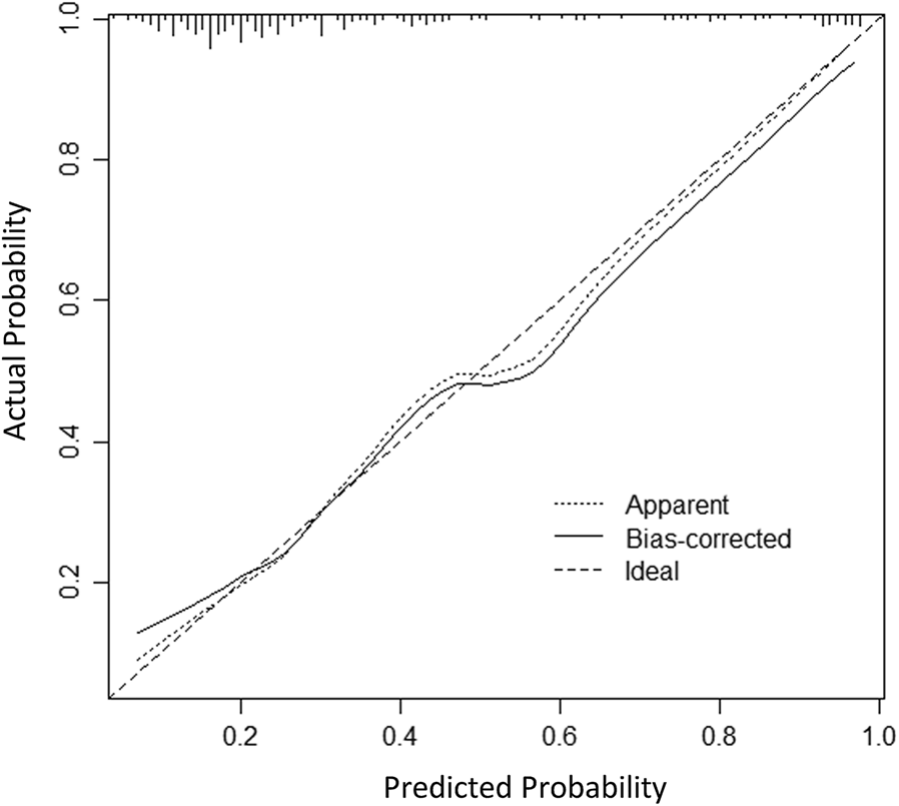
The calibration curve for predicting the risk of obstructive CAD in suspected CAD patients by an enhanced bootstrapping validation (500 bootstrap resamples) method. *CAD* coronary artery disease

The AUC calculated by enhanced bootstrapping validation (500 bootstrap resamples) was 0.785. Subsequently, a calibration curve (intercept = -0.106; slope = 0.843) was drawn to evaluate the calibration of the Model_3, which measures the relationship between the model’s predicted probability and the actual prevalence in the derivation cohort (Fig. 7). The 45° diagonal line indicates that the prediction exactly matches the actual, while a calibration curve above or below the 45° line indicates an underestimation or overestimation of patients’ risk.

## Discussion

The present study developed and validated a model based on rest-only ECG-gated SPECT MPI and cardiovascular risk factors to predict the risk of obstructive CAD. The AUC of the prediction model was 0.795 (95% CI: 0.741–0.843), which was better than the traditional models. The AUC calculated by enhanced bootstrapping validation (500 bootstrap resamples) was 0.785. The calibration curve (intercept = − 0.106; slope = 0.843) showed a good calibration of the model. The DCA showed that the constructed clinical prediction model had good clinical applications.

It is well known that stress SPECT MPI is a clinically accepted, evidence-based, non-invasive method for detecting myocardial ischemia. The typical manifestation of myocardial ischemia is abnormal stress perfusion (decreased perfusion or defect), and rest perfusion partially or completely returns to normal. In recent years, artificial intelligence algorithms such as deep learning have also been explored in stress or stress/rest MPI for diagnosing obstructive CAD [11, 20]. Essentially it was also a multivariate modeling process. The AUC of the deep learning model obtained by tenfold repeated testing was 0.83. However, there are many clinical patients who cannot undergo stress MPI due to various contraindications to stress tests. Taban Sadeghi et al. [6] found that only 30% of patients with suspected CAD presented with abnormal resting perfusion, with a low sensitivity. However, the sensitivity of resting perfusion combined with wall motion for the diagnosis of CAD improved to 46.8%, which is still low. Improving the accuracy of resting MPI for the diagnosis of obstructive CAD has important clinical implications. This study significantly improved the ability of resting MPI to diagnose obstructive CAD by combining clinical indicators, perfusion and myocardial function parameters to build a predictive model. This model may be a good complement to the limitations of clinical application of stress MPI.

In the process of variable selection, only SMS ≥ 2 and STS ≥ 2 among the myocardial function parameters were included in the model. This was not a coincidence. Wall motion abnormalities after stress have previously been reported to be a sensitive marker of severe CAD with a sensitivity of 78% and specificity of 85% [21]. Karimi-Ashtiani et al. [17] validated the incremental value of combined LV regional wall motion and thickening abnormalities in exercise stress SPECT MPI for detecting multivessel CAD compared to perfusion. Related mechanisms were explored in previous studies. In patients with multivessel disease, the degree of ischemia was underestimated due to relatively balanced global LV hypoperfusion without absolute myocardial regional blood flow quantification [22]. However, a 10–20% reduction in subendocardial blood flow is sufficient to induce severe regional wall dysfunction [23]. In addition, repeated myocardial ischemia in patient's daily life may lead to myocardial stunning, which manifests as normal perfusion with regional wall dysfunction [24–26]. The above-mentioned mechanisms explain the importance of myocardial function, such as wall motion, in the diagnosis of obstructive CAD. As classically reported, diastolic dysfunction may precede altered myocardial systolic function and represent an early step in the ischemic cascade. Multiple studies have also shown that evaluating diastolic function PFR has the added value of diagnosing CAD or myocardial ischemia [27–29]. In this study, the resting PFR was not included in the final model after LASSO regression selection, which may be related to the selection bias of the enrolled population or the automatic quantitative analysis process. Deeper mechanisms may require further research to explain.

The internal validation method used in this study was bootstrap resamples. The principle of bootstrap resamples is to use resampling technology to draw a certain number of samples from the original sample, allowing repeated sampling [19]. Each time the required statistic was calculated based on the sample drawn, and the variance of the statistic was obtained. Bootstrap resamples is a popular statistical method in modern statistics, especially in small samples [18]. Random split is a simple, easy to understand and implement internal validation method. At the same time, it requires a relatively large sample size. As the random state of the split changes, the accuracy of the model also changes, and the model cannot obtain a fixed accuracy.

In the latest ESC guidelines [30], computer tomography (CT) coronary angiography and MPI are Class I recommendations for patients with suspected CAD. CT coronary angiography is suitable for patients with contraindications to stress MPI. Previous study [31] has reported that the sensitivity and specificity of CT coronary angiography for diagnosing ACS in patients with low-risk chest pain were 82% and 92%. However, the AUC of the model constructed in this study was 0.795, demonstrating good discrimination, while different thresholds can be established according to clinical needs, with a trade-off between sensitivity and specificity. It is worth noting that patients with extensive coronary calcification, irregular heart rate, severe obesity, and inability to cooperate with breath-holding are not suitable for CT coronary angiography, due to reduced image quality. In addition, CT coronary angiography provides anatomical stenosis, while SPECT MPI can non-invasively obtain the relative myocardial perfusion information. Lewis et al. [32] reported that WM abnormalities detected by resting echocardiography were associated with severe (≥ 70%) coronary artery stenosis. Of the 77 abnormal wall motion regions, 60 (78%) were supplied by coronary arteries with ≥ 70% stenosis. However, echocardiography is dependent on the skill of the operator, which may vary between practitioners, and other parameters such as myocardial perfusion and wall thickening are not available with conventional echocardiography. Therefore, we believe that the predictive model based on rest-only ECG-gated SPECT MPI has more potential applications in future clinical practice.

## Limitation

Several limitations of this study should be considered. Firstly, no external validation was performed in this study, and its extrapolation will be further verified in the future. Second, all patients had contraindications to stress. Whether this nomogram can be applied to stress or stress/rest MPI patients needs further validation. Thirdly, the enrolled population included multiple stress contraindications and we did not perform subgroup analysis due to the number limitation. Other than that, the sample size included in this study was small and needed to be developed and validated in prospective, large sample, multicenter studies.

## Conclusions

This study shows that it is feasible to predict the prevalence of obstructive CAD by clinical factors, perfusion and myocardial function parameters obtained by rest-only ECG-gated SPECT MPI. In suspected CAD patients with contraindications to stress MPI, a nomogram combining factors including age, sex, SRS, SMS, STS, hypertension, diabetes and hyperlipidemia are able to calculate the risk of obstructive CAD, although further validation is needed.

## Data Availability

The datasets generated and/or analyzed during the current study are not publicly available due to departmental management, but are available from the corresponding author on reasonable request.

## Abbreviations

CAD: Coronary artery disease
SPECT: Single photon emission computerized tomography
MPI: Myocardial perfusion imaging
ECG: Electrocardiogram
PCI: Percutaneous coronary intervention
CABG: Coronary artery bypass grafting
LAD: Left anterior descending coronary artery
LCX: Left circumflex coronary artery
RCA: Right coronary artery
SRS: Summed rest score
SMS: Summed motion score
STS: Summed thickening score
EDV: End-diastolic volume
ESV: End-systolic volume
LVEF: Left ventricular ejection fraction
PFR: Peak filling rate
